# Organ Donation Conversations on X and Development of the OrgReach Social Media Marketing Strategy: Social Network Analysis

**DOI:** 10.2196/59872

**Published:** 2025-02-06

**Authors:** Wasim Ahmed, Mariann Hardey, Josep Vidal-Alaball

**Affiliations:** 1 Business School, University of Hull Hull United Kingdom; 2 Business School, Durham University Durham United Kingdom; 3 Unitat de Recerca i Innovació, Gerència d'Atenció Primària i a la Comunitat de la Catalunya Central, Institut Català de la Salut Manresa Spain; 4 Intelligence for Primary Care Research Group, Fundació Institut Universitari per a la Recerca a l'Atenció Primària de Salut Jordi Gol i Gurina Manresa Spain; 5 Department of Medicine, Faculty of Medicine, University of Vic Vic Spain

**Keywords:** organ donation, organ transplant, social media, health, social network analysis, marketing strategy, awareness, public health, health information, qualitative, thematic analysis, NodeXL Pro, algorithm, elite tier, digital health, United Kingdom, X

## Abstract

**Background:**

The digital landscape has become a vital platform for public health discourse, particularly concerning important topics like organ donation. With a global rise in organ transplant needs, fostering public understanding and positive attitudes toward organ donation is critical. Social media platforms, such as X, contain conversations from the public, and key stakeholders maintain an active presence on the platform.

**Objective:**

The goal is to develop insights into organ donation discussions on a popular social media platform (X) and understand the context in which users discussed organ donation advocacy. We investigate the influence of prominent profiles on X and meta-level accounts, including those seeking health information. We use credibility theory to explore the construction and impact of credibility within social media contexts in organ donation discussions.

**Methods:**

Data were retrieved from X between October 2023 and May 2024, covering a 7-month period. The study was able to retrieve a dataset with 20,124 unique users and 33,830 posts. The posts were analyzed using social network analysis and qualitative thematic analysis. NodeXL Pro was used to retrieve and analyze the data, and a network visualization was created by drawing upon the Clauset-Newman-Moore cluster algorithm and the Harel-Koren Fast Multiscale layout algorithm.

**Results:**

This analysis reveals an “elite tier” shaping the conversation, with themes reflecting existing societal sensitivities around organ donation. We demonstrate how prominent social media profiles act as information intermediaries, navigating the tension between open dialogue and negative perceptions. We use our findings, social credibility theory, and review of existing literature to develop the OrgReach Social Media Marketing Strategy for Organ Donation Awareness. The OrgReach strategy developed is based on 5 C’s (Create, Connect, Collaborate, Correct, and Curate), 2 A’s (Access and Analyse), and 3 R’s (Recognize, Respond, and Reevaluate).

**Conclusions:**

The study highlights the crucial role of analyzing social media data by drawing upon social networks and topic analysis to understand influence and network communication patterns. By doing so, the study proposes the OrgReach strategy that can feed into the marketing strategies for organ donation outreach and awareness.

## Introduction

### Background

The current shortage of organ transplants necessitates fostering public understanding and positive attitudes toward organ donation. The critical need for organ donation in the United Kingdom is further highlighted by the staggering number of patients currently waiting for a transplant, which stands at 7489 at the time of writing [1]. Social media, as a crucial platform for public health discourse [2], can harbor topics like organ donation. While research has explored increasing donor rates, a gap exists in understanding how online information environments shaped by prominent voices influence public perceptions. This study addresses this gap by using social network analysis (SNA) to examine conversations about organ donation on X (formerly Twitter). Specifically, we investigate how prominent social media profiles and users seeking health information influence these conversations and the types and forms of advocacy.

While previous research has examined the link between public attitudes and external factors like health care environments [3-5], we shift the focus inward, investigating how individuals navigate and interact with organ donation information on the internet. This approach offers insights into donation practices and the development of commercial organizations in this space. Specifically, we use SNA on platform X to map connections and influence patterns among the most prominent user profiles during the initial stages of information-seeking related to organ donation. Our analysis focuses on three key areas: (1) identifying users occupying pivotal positions within the network, (2) examining the emergence and persistence of an “elite tier” as the network evolves, and (3) exploring thematic and topical trends across active user accounts and dedicated health organizations.

This study defines the “elite tier” as a group of highly influential users within the organ donation community on social media. These individuals are distinguished by their ability to shape information flow, disseminate knowledge, and mobilize social media discourse. We identify elite-tier members based on their influence on their network (as measured by their betweenness centrality). We use our findings to develop the OrgReach Social Media Marketing Strategy for Organ Donation Awareness.

### Examining the Credibility of Organ Donation Information on Social Media

While credibility in social media contexts has received significant attention within social science, media and cultural studies, and internet health literacy research, a critical gap remains in understanding its dynamic nature. This gap specifically concerns how credibility evolves and shapes how individuals navigate and engage with health information on social media. For example, Alikunju and Sulochana [6] argue that authenticity and network visibility constitute the core elements of credible health information on the internet. We use credibility theory [7] to explore the construction and impact of credibility within social media contexts in organ donation discussions. Specifically, we are interested in understanding the frequency and occurrence of credible sources of information (ie, health care professionals or organ donation organizations) compared to less credible sources of information. We also aim to explore the impact of credible actors on network reach on social media platforms.

### Related Work

Previous research has studied organ donor registration on Facebook [8] and how the platform allowed users to specify they were organ donors. The study found that the Facebook intervention dramatically increased Organ Donation registration within the United States. The study highlights the potential of social media platforms in increasing organ donation awareness on social media. Other studies have investigated the ethical challenges in the use of social media in relation to privacy and confidentiality, pointing to the fact that there are no national ethical guidelines in the United States in relation to social media use for fostering organ transplantation [9]. Other research has examined different approaches to promoting kidney donation using social media data [10], to examine the role of Twitter for organ donation awareness [11], and to identify opinion leaders on Weibo [12].

Although social media platforms have been around since the early 2000s, using PubMed, we find that the first papers specifically examining organ donation and social media (as reflected in their title) appeared to have been published in 2012. The first outputs were related to a large-scale study that examined how university students could use social media to generate awareness and support for organ donation [13] alongside a published case report [14]. Of the 17 papers PubMed located published between 2012 and 2024, the most appeared to be published in 2023 (n=4). Moreover, previous research has analyzed the change in attitude toward organ donation following an educational program [15] where teenagers disseminate content on social networks to raise societal awareness. However, to the best of our knowledge, we found no other studies that aimed to perform a topic identification-driven SNA of X data that developed a specific marketing strategy for organ donation awareness.

The overall aim of this study is to identify the topics discussed on X relating to organ donation, to identify popular content, and to develop a specific marketing strategy for organ donation awareness.

## Methods

### Key Assumptions

The “Methods” and “Results” sections of this study frequently mention X users within the network, which are our “nodes,” a concept from SNA. When we refer to “edges,” these are our connections between users and can include reposts, mentions, and replies to users. Additionally, the study uses “betweenness centrality” as a metric to gauge a user’s influence within the network. This measures the extent to which a node influences the flow of information among other nodes. This study follows the methodological approach from previous research [16-18] applied to the topic of organ donation.

### Stage 1: Data Retrieval

Data were retrieved from X mostly between October 2023 and May 2024, covering a 7-month period. NodeXL Pro was used to retrieve data using the search string “organ donation.” This would retrieve any post that contained “organ donation” or who replied to, mentioned, reposted, or quoted in those posts. It is important to note that content was retrieved from X without limiting it to a specific country. However, platform demographics such as more US users will mean that our sample is likely to contain more content from English-speaking countries. This is important as there will be differences in responses across different countries and also because of differences between opt-in and opt-out systems.

### Stage 2: Data Preparation

Once data were retrieved, there were 20,124 users within the network and a total of 33,830 posts. These data were processed in NodeXL Pro (version 1.0.1.530) for further analysis. Within the network visuals, the edge colors are based on edge weight values. The edge widths were based on edge weight values. The vertex sizes (ie, the users) were based on in-degree values.

### Stage 3: Data Analysis

The SNA method was used to analyze the data conducted in NodeXL Pro. The graph’s vertices were grouped by cluster using the Clauset-Newman-Moore cluster algorithm [19]. The graph was laid out using the Harel-Koren Fast Multiscale layout algorithm [20]. We used thematic analysis [21] to develop themes and subthemes. After examining the groups through SNA, it was found that discussions within each group are multifaceted and are not confined to a single topic per group, but a multitude of conversations are taking place. Therefore, an analysis was conducted on the most shared content within the network overall. The 300 most popular posts (identified by finding the posts most reshared) had a total reshare value of n=3,975,866 with a total of n=6,398,623 favorites. We used a thematic analysis [21] of the top 300 posts until thematic saturation occurred. This thematic analysis involved a team-based coding approach where each post was assigned codes/labels by one author and then discussed and refined by the wider team. The labeling and analysis took place within Excel (Microsoft Corp). This allowed us to identify the most popular themes of discussions across the groups.

### Ethical Considerations

This study examined data only in aggregate and no individuals who are not already in the public domain are mentioned or named in this study. Moreover, this study does not quote or use any content from any individuals, and only publicly shared information on the X platform is captured and analyzed. Ethical approval for this study falls under the institutional review board of Newcastle University (26055/2022). During the editing process, we drew upon generative language models (GPT-4o) and Grammarly to improve readability and identify typographical errors of existing human-authored text with oversight from the authors, but these models were not used to generate any content of their own. This was particularly useful as supported an author who is dyslexic.

## Results

### SNA and Qualitative Interpretation

**Figure 1:**
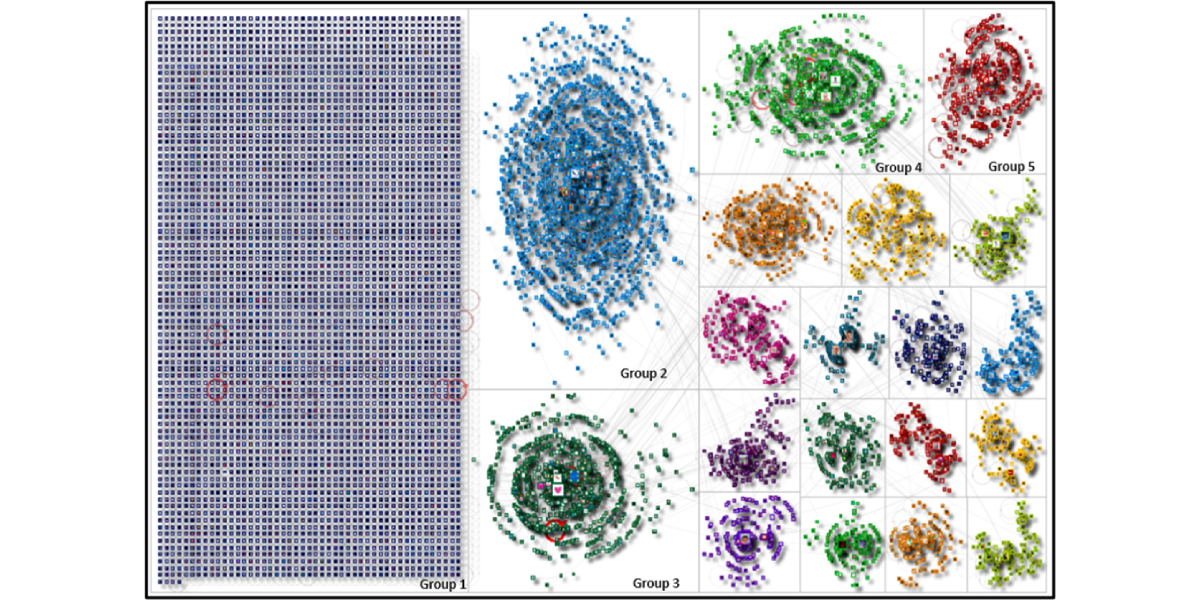
Social network visualization of the organ donation X network (October 2023 to May 2024).

Figure 1 shows a visual overview of how users connect with each other on X when conversing about organ donation and shows the top 20 groups. The network visual is simply a representation of groups that have formed based on user interactions (resharing, reposting, and replying to users). The largest group in the network (group 1) is an isolated group where users post without mentioning other users. There is one large community cluster (group 2) followed by three medium-sized community clusters (groups 3-5). The remainder of the groups (groups 6 to 20) consist of smaller community clusters.

This analysis tells us that many individual posts are sent from users about organ donation (as noted from group 1), which include advocacy and stories. It also tells us that there are vocal conversations taking place by users who have formed their own communities and that some large, medium, and small communities on X are actively conversing about organ donation. The network visual also reveals that there is little interactivity between the groups.

The main themes and their subthemes from this analysis are presented below.

### Theme 1: Ethical and Legislative Framework

#### Ethical Considerations and Debates

Users would discuss ethical concerns around organ donation, which would include mandatory donation and the use of human tissues in research.

#### Legislative and Policy Measures

Users would also converse about organ donation laws, transplantation regulations, and policies such as opt-out systems.

#### Controversy and Public Opinion

Users would also comment on controversial cases of organ harvesting and make claims that were not verifiable and potentially misinformation. Users would also hold debates and have critical discussions about organ donation.

### Theme 2: Awareness, Advocacy, and Engagement

#### Awareness and Advocacy in Diverse Communities

Conversations by users included raising awareness of organ donation in minority communities and promoting global advocacy.

#### National and Cultural Engagement

Users would also discuss incorporating organ donation into national and cultural events to boost community involvement.

#### Impact of Public Figures and Influencers

Conversations also included the use of public figures and celebrities to advocate for organ donation and share their personal stories.

### Theme 3: Recognition and Community Support

#### Recognition and Honor for Donors

Discussions included official recognition and public ceremonies for organ donors and their families.

#### Community Solidarity and Support

Within this subtheme, there were conversations and demonstrations of community support and consensus regarding organ donation efforts.

### Theme 4: Personal Impact and Health Care Achievements

#### Inspirational Stories and Acts of Heroism

Within this subtheme, there were also personal accounts of donors who had made significant contributions by donating organs, and their stories were used to inspire others.

#### Health Care Achievements and Milestones

There were users, including health care professionals, who would celebrate key successes in organ donation and transplantation. These posts would also highlight positive recipient outcomes.

### Additional Findings

Table 1 provides a ranking of the top 10 users posting on X within the network around organ donation. These users were defined as “elite tier” users as noted in the introduction due to their influential position within the network as measured by betweenness centrality.

The most influential user was the official NHS (National Health Service) organ donation account in the United Kingdom. Their user bio suggests active engagement with the public to improve and save lives through organ donation. They note that they respond to posts during specific hours, indicating a personalized approach to community interaction.

The second rank is held by the PMO (Prime Minister’s Office) India, which is the Office of the Prime Minister of India. This account’s high centrality score reflects its influence and broad reach in disseminating information related to organ donation within India. The account posted positive comments about organ donation, reaching a wide audience in India.

The third most influential user was Justin Trudeau, the 23rd Prime Minister of Canada. Trudeau’s user bio is presented in English and French, highlighting bilingual communication for broader outreach within Canada. Justin Trudeau has been a vocal supporter of organ donation on the X platform in the past, and the account appeared to be tagged into various conspiracies and potential mis and disinformation networks.

Ranks 4 through 10 were held by individual users worldwide, whom we categorized simply as “Citizens.” Some users had specific interests, such as a business-focused user and a heart transplant recipient. Of the users who disclosed their location, users derived from Canada, the United States, and the United Kingdom. This highlights that ordinary citizens with modest followings can hold significant positions within the network, contributing to discussions, spreading awareness, or advocating for organ donation.

Table 2 provides the top “domains” that users X posts were linking to. A domain analysis allows us to detect popular web pages. Table 2 highlights a wide-ranging effort to promote organ donation information across different types of websites, from government and health services to news media and social platforms. Link shorteners like bit.ly and ow.ly indicate a focus on social media sharing and tracking engagement with this content. The top-ranking domain, transplantnews.com, suggests a dedicated source of information that will likely serve as a hub for those interested in the subject. These domains are further discussed below.

Discussing these, starting from the most popular web domain, we can see that the transplantnews.com domain is the most frequent linker to organ donation content (n=736). It is a specialized website focused on news related to organ transplants. The second rank is the Indian government’s official domain, gov.in (n=311), which indicates a significant amount of content or references related to organ donation within the Indian government’s online resources. In third place is dlvr.it (n=310), a content delivery network often used to manage and disseminate information across various platforms, suggesting widespread sharing of organ donation-related content.

**Table 1:** Top 10 influencers on X related to organ donation (October 2023 to May 2024).

Rank	Userhandle (anonymized if citizen)	Name	User bio	Location	Betweenness centrality
1	nhsorgandonor	NHS^a^ Organ Donation	Saving and improving lives. Official account for the NHS Organ Donor Register. We love reading your tweets and reply to them between 0700-2300 #OrganDonation	United Kingdom	12713160.850
2	pmoindia	PMO^b^ India	Office of the Prime Minister of India	India	9036848.857
3	justintrudeau	Justin Trudeau	Father, @liberal_party Leader, 23rd Prime Minister of Canada. | Papa, chef du @parti_liberal 23e premier ministre du Canada.	Canada	8986407.605
4	Citizen	Anonymized	Anonymized	Undisclosed	8105094.910
5	Citizen	Anonymized	Anonymized	Canada	7750991.313
6	Citizen	Anonymized	Anonymized	Undisclosed	7333731.145
7	Citizen Business Interest	Anonymized	Anonymized	United States	7301905.351
8	Citizen	Anonymized	Anonymized	Undisclosed	7215205.365
9	Citizen Political Influnfcner	Anonymized	Anonymized	United States	6379487.669
10	Citizen Heart Transplant Recipient	Anonomyized	Anonomyized	United Kingdom	6185548.502

^a^NHS: National Health Service.

^b^PMO: Prime Minister’s Office.

**Table 2:** Top 10 domains on X related to organ donation (October 2023 to May 2024).

Rank	Top domains	Count, n
1	transplantnews.com	736
2	gov.in	311
3	dlvr.it	310
4	bit.ly	281
5	youtu.be	150
6	facebook.com	140
7	nm-4.com	128
8	ow.ly	109
9	nhs.uk	103
10	co.uk	98

In fourth place, we have bit.ly (n=281), a URL-shortening service. The relatively high count indicates that many shortened URLs linking to organ donation content have been created, highlighting a general trend of concisely disseminating this important information across different platforms. This practice likely enhances user accessibility and simplifies the process of sharing such links.

The fifth-ranked domain is youtu.be (n=150), the shorthand for YouTube signifies a moderate level of engagement with video content related to organ donation. This number reflects the efforts made to use multimedia platforms to raise awareness and educate the public about organ donation through videos, which can be a compelling way to convey the impact and importance of this act. Following that, facebook.com stands in the sixth position (n=140). As a major social media platform, Facebook’s role in spreading organ donation content is important. Although it ranks lower than some other domains, the figure indicates that organ donation discussions and information sharing are part of the social conversation on Facebook.

The domain nm-4.com is listed in seventh place (n=128), the short version of the full URL which points to an official website belonging to the Indian Prime Minister, this web page shared information about Narendra Modi’s support toward organ donation. Ranking eighth is ow.ly (n=109), another URL-shortening service akin to bit.ly. Its use for organ donation content implies that there is an active effort to track engagement and reach of shared links on various social media and platforms on the internet. The ninth position is held by nhs.uk (n=103), the UK National Health Service’s official domain. This domain’s contribution highlights the UK health authority’s commitment to educating and providing resources on organ donation to the public, reflecting the importance of institutional support in promoting health initiatives.

Finally, co.uk (n=98) encompasses a range of UK-based websites. This generic top-level domain’s appearance in the top ten signifies that various UK entities collectively discuss and link to organ donation content. This points to a country-wide effort to endorse organ donation awareness and registration.

Table 3 provides an overview of the top 10 hashtags that users were using. In first place was the hashtag #organdonation (n=5413), which is the most widely used hashtag concerning organ donation discussions. The second most prevalent hashtag is #donatelife (n=1361). This hashtag resonates with the community’s call to action for individuals to contribute to the cause of saving lives through organ donation. In third place is the hashtag #giftoflife (n=974). This hashtag emphasizes organ donation’s life-saving and life-enhancing benefits, celebrating it as an altruistic gift a person can offer others. The fourth most popular hashtag was #organtransplant (n=867). This hashtag is likely used to share information, personal stories, and developments related to the organ transplant process.

In fifth place was #tissuetransplant (n=756), which points to discussions specific to the transplantation of tissues, another crucial aspect of the broader conversation on transplantation and organ donation. In sixth place was the hashtag #transplantnews (n=742), suggesting a focus on sharing news and updates related to transplant medicine and stories of individuals involved in the transplant process.

The seventh-ranked hashtag is #organtissue (n=478), and it brings to light the conversation about both organ and tissue donation, indicating a combined discourse that highlights the need for donations of all types of body tissues. The hashtag #kidneytransplant (n=359) takes the eighth spot, denoting a specific focus within the organ donation community on kidney transplants, reflecting the high demand and frequency of this type of transplant. The hashtag #transplant (n=339) was in ninth place. This more general hashtag indicates a broad discussion encompassing the entire spectrum of transplant-related topics. Finally, the hashtag #savelives (n=315) occupied the tenth position. This compelling and impactful call to action highlights the ultimate goal of organ donation: to save and improve lives.

**Table 3:** Top 10 hashtags on X related to organ donation (October 2023 to May 2024).

Rank	Top hashtags	Count, n
1	organdonation	5413
2	donatelife	1361
3	giftoflife	974
4	organtransplant	867
5	tissuetransplant	756
6	transplantnews	742
7	organtissue	478
8	kidneytransplant	359
9	transplant	339
10	savelives	315

### Development of the OrgReach Marketing Strategy

#### Overview

We draw upon our results, our review of existing research [8-12], and social credibility theory [7] to develop the OrgReach Marketing Strategy. Specifically, we draw upon our results around:

- The nature of the network and the lack of cross-group conversations that were found in our SNA.
- Our insights into the opinion leaders within the network and their characteristics.
- The characteristics of the most popular posts that were sent in terms of retweets and favorites and the types of content that attracted reposts.
- The most popular web domains within the network in terms of link clicks and the types of studies that they were sharing.

We conceptualize the 5 C’s (Create, Connect, Collaborate, Correct, and Curate), 2 A’s (Access and Analyse), and 3 R’s (Recognize, Respond, and Re-evaluate), which we name the OrgReach Marketing Strategy. Each element is further outlined below.

#### The 5 C’s

##### Create

Develop engaging and educational content related to your topics of interest. Emotional content, including images or video-based testimonials highlighting organ donation’s need and impact on lives, may gain more visibility. To assess the appeal and educational effectiveness of content, organizations should continuously monitor engagement metrics (likes, shares, comments, and reach) to determine the popularity and engagement with content. Moreover, direct qualitative feedback from users and A/B testing of content could help provide further evaluation of content.

##### Connect

To build a strong community and foster conversations and connections between individuals, health care professionals, and advocacy groups. Using the search feature to find similar organizations and conversations and break down conversational silos on social media. To assess and evaluate whether organizations are succeeding in their network building, they can determine their social network metrics (network density, clustering, and cross-group interactions). Moreover, tracking follower and advocacy accounts, as well as user surveys, could help evaluate the success of connection-building.

##### Collaborate

Partner with influencers, health care organizations, and governmental bodies to collaborate on content creation, amplify the message, and reach a wider audience. Codevelop and share content and incorporate other “@” tags within posts. More credible influencers will likely lead to further impact. Organizations can select partners such as health care organization and governmental bodies based on the alignment of values and for influencers, their reach, and audience. More sophisticated analyses, such as engagement quality, influencer identification, and credibility assessments, can also be drawn upon in this phase to identify the quality and consistency of collaborative content.

##### Correct

Monitor and address any misinformation users share, ensuring the public receives accurate and trustworthy information about organ donation. Corrected information may lead to your organization’s further visibility. Organizations can draw upon text-analysis tools, such as NodeXL Pro, introduced earlier, and this could help identify high-frequency keywords that can highlight the presence of misinformation. This analysis can be supplemented with sentiment and web-domain level analysis where the credibility of websites is assessed. Moreover, organizations can look to existing fact-checking resources and encourage community reporting of misinformation.

##### Curate

In addition to original content, organizations can handpick and share relevant stories, news, and data relevant to organ donation to keep the audience informed and engaged. Organizations can set up news monitoring on platforms such as Google News for relevant, credible content that can include stories that resonate emotionally with the target audience. Organizations should ensure content across diverse perspectives, including minority communities and different cultural approaches to organ donation. Organizations can also incorporate feedback loops, which provide social media followers to provide feedback on content that is shared.

#### The 2 A’s

##### Access

Ensure that the information you share is accessible to everyone, removing barriers, that is, a public-facing social media account with an easy-to-find user handle. This further enhances credibility.

##### Analyze

Use SNA and credible influencer detection to understand audience behavior and refine strategies for better engagement and impact. Using this analysis and output of key users and content to feed back into the 5 C’s.

#### The 3 R’s

##### Recognize

Publicly acknowledge and celebrate the altruism of donors and the success stories of recipients to inspire others.

##### Respond

Establish a reliable and responsive communication channel through social media to answer queries and engage with the audience in real time.

##### Reevaluate

Continuously assess the strategy’s effectiveness and make data-driven adjustments to improve outreach and advocacy efforts.

## Discussion

### Principal Findings

Previous research has studied the potential of social media platforms for increasing donor registrations and strategies for sharing organ donation-related content. This study contributes to the literature by providing insights into users’ conversations around organ donation, identifying influencers, and uncovering the network structure of the elite tier and key URLs and hashtags. These findings were then used to develop the OrgReach Marketing Strategy to offer practical suggestions and strategies for organizations in this area. Our findings and their broader implications will be further discussed.

In our analysis, the structure of user interactions on X through SNA provided insights into the shape of the discourse surrounding organ donation. The largest group was where users would send isolated posts, suggesting that individuals share personal stories and advocacy messages independently. Large and smaller interactive community clusters indicated that more engaged discussions were also occurring, which are important for fostering support among users considering organ donation. The limited interactivity between the groups suggests the potential for enhancing dialogue across different clusters to unite advocacy efforts and enhance the conversation around organ donation. By understanding these interactions, stakeholders could better tailor their communication strategies to increase awareness and participation in organ donation. This would allow stakeholders to maximize the role of social media as a tool for impactful advocacy.

Our analysis also revealed that a wide array of topics related to organ donation were discussed. These ranged from ethical debates and legislative discussions to advocacy efforts and personal impact stories. This study found that users engage in conversations about the ethical implications of mandatory donation, the use of tissues in research, and the effects of laws and opt-out systems. Controversial topics often include misinformation, which highlights the need for accurate communication. Advocacy through public figures and cultural events boosted awareness within the network, especially in minority communities. The recognition of donors and community support on X emphasized organ donation’s societal value and inspirational potential.

The NHS Organ Donation account, a significant member of the elite tier, actively engages with the public through structured interaction hours, underscoring its commitment to saving lives. This highlights a strategic approach to online health communication, which may, in part, have led to the greater impact of the account. Influential leaders such as the PMO India and Canada’s Prime Minister Justin Trudeau show how political figures can leverage their platforms to significantly impact public discourse on health issues. However, political figures can also be targeted for misinformation, as was the case with Trudeau’s account. Moreover, the presence of anonymized citizens and public figures from diverse locations like the United Kingdom, Canada, and the United States among the top influencers indicates that organ donation discussions attract a wide range of contributors, from individuals sharing personal experiences to those with business or political interests. This provides a variety of viewings being shared.

The analysis of the top domains linked in these discussions revealed a strategic use of diverse online resources to promote organ donation. These included specialized news outlets such as transplantnews.com, as well as governmental and social media platforms. The variety in content distribution channels highlights the concerted efforts to maximize outreach and engage different audiences effectively. URL shorteners and content delivery networks highlight a sophisticated approach to content dissemination, ensuring that information reaches a broad audience efficiently and is easily shareable.

The analysis and identification of popular web domains, hashtags, and the development of the OrgReach Marketing Strategy aim to contribute to existing literature and provide practical suggestions and strategies for social media engagement and outreach.

### Limitations and Future Research Agenda

This study focused on retrieving data from X (formerly Twitter). Therefore, our empirical findings would not capture discussions on other platforms like Facebook, Instagram, or specific Web forums. Future research could examine content across various platforms to provide a critical comparative perspective. Moreover, this study examines a single time slice and captures conversations across a specific 7-month period, and content may differ across other periods. Future research could retrieve and compare different periods to identify changes in influencers and conversations. Future research could also seek to test and validate the proposed OrgReach Social Media Marketing Strategy in real-world campaigns which would help to strengthen the practical applicability of the findings.

Moreover, it is important to note that this study examined content aggregately and did not stratify different countries. This is because certain countries operate under an “opt-in” to organ donation system, such as the United States, where campaigns typically emphasize individual registration. In opt-out countries, campaigns focus on promoting organ donation’s altruistic and societal benefits to encourage families to make informed decisions. Based on these differing contexts, the study does not differentiate social media content or user interactions. The OrgReach Marketing Strategy could also be enhanced by incorporating specific guidance for campaigns tailored to these legislative frameworks.

## Abbreviations

NHS: National Health Service
PMO: Prime Minister’s Office
SNA: social network analysis

## Data Availability

The data sets generated during or analyzed during this study are available from the corresponding author upon reasonable request.
